# Production of Ultracold XOH (X = Ca, Sr, Ba) Molecules by Direct Laser Cooling: A Theoretical Study Based on Accurate Ab Initio Calculations

**DOI:** 10.3390/molecules30091950

**Published:** 2025-04-28

**Authors:** Jingbo Wei, Peng Li, Jizhou Wu, Yuqing Li, Wenliang Liu, Yongming Fu, Jie Ma

**Affiliations:** 1 State Key Laboratory of Quantum Optics Technologies and Devices, Institute of Laser Spectroscopy, College of Physics and Electronic Engineering, Shanxi University, Taiyuan 030006, China; 2 Collaborative Innovation Center of Extreme Optics, Shanxi University, Taiyuan 030006, China

**Keywords:** ab initio calculations, triatomic molecule, direct laser cooling, transition channel, Franck–Condon factors

## Abstract

Effective laser cooling schemes are fundamental for preparing ultracold triatomic molecules. Here, efficient laser cooling strategies for alkaline-earth hydroxides (XOH, X = Ca, Sr, Ba) are proposed using high-precision quantum calculations. By mapping Λ-S- and Ω-state potential energy surfaces, we identified quasi-closed optical cycles with dominant Franck–Condon factors (FCFs) and strong transition dipoles. The scheme utilizes targeted repumping to suppress vibrational leaks, enabling >10^4^ photon scatters per molecule, exceeding Doppler cooling requirements. These results establish XOH molecules, particularly BaOH, as viable candidates for laser cooling experiments, providing key theoretical insights for ultracold triatomic molecule production.

## 1. Introduction

Polyatomic molecules have become popular laser direct cooling targets due to their unique properties such as multiple permanent dipole moments, a long coherence time, and a large count rate, which provide a new high-quality platform for the development of quantum science and technology [1,2,3,4,5]. However, due to the complexity of their structures and the rapid internal relaxation and dissociation mechanisms, the energy levels are prone to rapid decay, and it is difficult to find reasonable transition channels, which makes the preparation and manipulation of ultracold triatomic molecular gases extremely challenging.

M–OH species are classified as triatomic molecules due to their covalent bonding structure. However, their open-shell electronic configuration—featuring a single unpaired electron in the metal-centered σ orbital (with a ground state of ^2^Σ^+^)—endows them with radical-like characteristics that are critical for spectroscopic detection and gas-phase kinetics. These molecules have been extensively studied owing to the transition properties associated with their unpaired electrons. In 2016, equilibrium structures and FCFs of linear polyatomic molecules were obtained via quantum chemical computational methods by Isaev et al. [6], while the cooling possibilities of nonlinear and chiral molecules were evaluated. Subsequently, laser cooling of polyatomic molecules containing heavy elements was similarly investigated [7]. The field of laser cooling has recently expanded to include heavy-element-containing molecules and more complex polyatomic systems. Notably, radium hydroxide (RaOH) has been identified as a suitable candidate for laser cooling through calculations of its transition dipole moments, parity-odd (P-odd) properties—which reverse signs under parity transformation, indicating parity symmetry violation—and parity- and time-reversal-odd (P-, T-odd) properties, characterized by sign changes under combined parity- and time-reversal transformations. For the first time, Symmetry Breaking and CP Violation are calculated for a triatomic molecule with open electronic shells. In 2019, the hybridization and spin structures of YbCCCa and YbCCAl molecules were calculated, establishing their importance for laser cooling applications. The feasibility of their precision measurement was also investigated, providing a tool for the experimental manipulation of metal species [8]. In 2020, the laser cooling of asymmetric gyroscopic molecules was extensively investigated by Augenbraun et al. [9]. The ab initio calculation method was extended to nonlinear asymmetric molecules, while the effect of the discrete breaking of the symmetry structure of triatomic molecules on laser cooling was analyzed. A route applicable to the laser cooling of such molecules was proposed by multiple optical cycling centers (OCCs) and may be more beneficial for laser cooling, as noted by Kotochigova et al. [10]. Laser cooling of large organic molecules was discussed by Ivanov et al. [11]. It was proposed that aromatic ligands such as benzene can have multiple cycling centers, which opens new routes in the field of quantum information. In 2021, the effect of organic ligand functionalization on the FCF was assessed by Dickerson et al. [12]. Chemical substitution-enhanced optical cycling provides a design principle for quantum information and precision measurement systems. By leveraging Hammett constants to guide electron-withdrawing substituent selection, this approach counteracts the detrimental effects of added vibrational modes on optical cycle closure. Theoretical calculations show that chemical substitution promotes optical cycling, which opens new paths for laser cooling of large molecules. In the same year, the functionalization of optical cycling ligands for very large molecules that can form planes was studied, and calculations show that optical cycling can be retained on arenes [13].

Related experimental and spectroscopic studies are also developing rapidly [14,15,16,17]. SrOH [18,19,20] (~750 μk), CaOH [21,22], YbOH [23], and CaOCH_3_ [24] have been successively cooled by the Doyle group for direct laser cooling. The FCF is also measured and predicts a laser cooling method for molecules with lower symmetry such as chiral molecules. The laser spectroscopy of YbCH_3_ [25] was performed by Augenbraun et al. The vibrational frequencies, vibrational branching ratios, and radiative lifetimes are measured and assessed for their feasibility for laser cooling and precision measurements.

Polyatomic molecules containing heavy nuclei have garnered significant interest for novel physics investigations beyond the Standard Model [26,27,28], as well as applications in cold chemistry, photochemistry, and quantum information science. Specifically, their electronic structure exhibits advantageous characteristics for precision measurements, particularly the quasi-diagonal nature of Franck–Condon matrices between ground and excited electronic states. This property arises from the enhanced diffuse valence electronic orbital compared to their lighter homologs, thereby enhancing their potential for studies requiring strong state-selective transitions. The relativistic expansion of valence orbitals in heavy elements facilitates this distinctive electron delocalization, creating favorable conditions for manipulating quantum states and probing fundamental symmetries.

To date, while a greater number of polyatomic molecules have been directly cooled by lasers, the cooling schemes are complex and focused on the transition between the ground state and the first excited state. Further possibilities for laser cooling of polyatomic molecules will be provided by studying the nature of the transition and the spontaneous radiation correlation between the higher electronic states and the ground state. In this work, we carried out studies of the electronic structure, the nature of the transition, and the orbital occupation by high-precision ab initio calculations for XOH (X = Ca, Sr, Ba). The potential energy surface of the molecules, the direct laser cooling scheme, and the electronic occupation are obtained, while the feasibility of the cooling scheme is analyzed.

## 2. Results

### 2.1. Potential Energy Curves

At short bond lengths, the electronic states of XOH (X = Ca, Sr, Ba) are predominantly characterized by a limited number of configurations involving single excitations of the unpaired electron. However, at large bond lengths, the electronic states undergo a pronounced character transformation, requiring configurations with three or more open shells to adequately describe the system [29]. Laser cooling of XOH molecules primarily occurs near the equilibrium bond length. In this work, particular emphasis is placed on investigating potential energy curves (PECs) at shorter bond lengths through systematic theoretical calculations.

**Figure 1:**
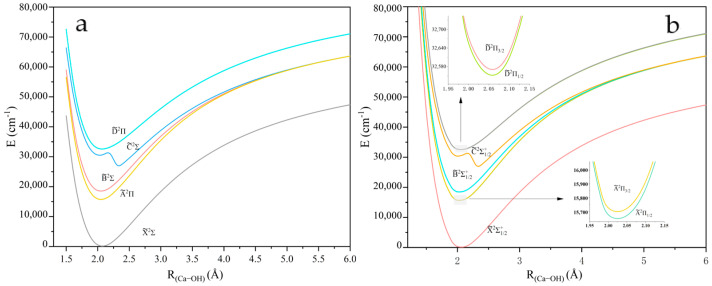
(**a**) PECs of the linear CaOH with O−H fixed at 0.952 Å, where panel (**b**) presents the results considering the spin–orbit coupling effect.

**Figure 2:**
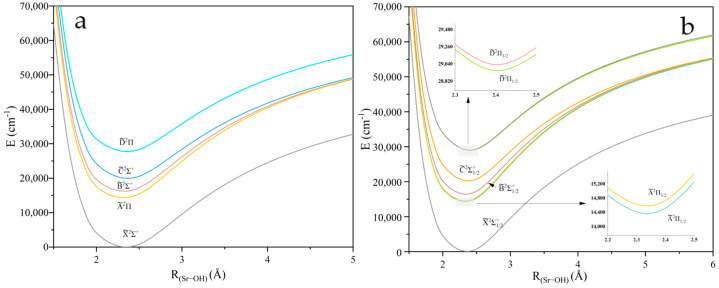
(**a**) PECs of the linear SrOH with O−H fixed at 0.951 Å, where panel (**b**) presents the results considering the spin–orbit coupling effect.

**Figure 3:**
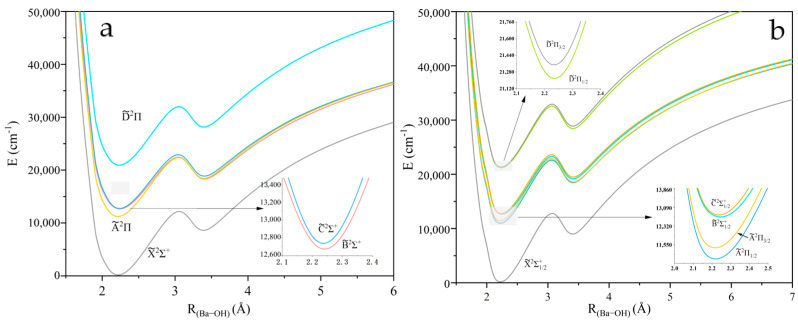
(**a**) PECs of the linear BaOH with O−H fixed at 0.959 Å, where panel (**b**) presents the results considering the spin–orbit coupling effect.

The PECs of five Λ-*S* states of XOH investigated near the equilibrium nuclear spacing at the MRCI + Q level of theory are shown in Figure 1, Figure 2 and Figure 3. The calculations for all molecules contain five electronic states: ${\tilde{X}}^{2}\Sigma^{+}$, ${\tilde{A}}^{2}\Pi$, ${\tilde{B}}^{2}\Sigma^{+}$, ${\tilde{C}}^{2}\Sigma^{+}$, and ${\tilde{D}}^{2}\Pi$. The PECs consider spin–orbit coupling effects, where the ^2^Π states split into Ω = 1/2 and Ω = 3/2 states. The spin–orbit splitting Λ for the ^2^Π states is 51.99/19.51 cm^−1^, 206.6/77.15 cm^−1^, and 444.09/126.38 cm^−1^ for CaOH, SrOH, and BaOH, respectively. The local minimum observed in the BaOH potential energy curve at ~3.4 Å (Figure 3) can be attributed to the interaction between ionic and covalent states, a phenomenon less pronounced in CaOH and SrOH due to the smaller size of the alkaline-earth metal. This feature highlights the importance of considering relativistic effects and state mixing in heavy triatomic molecules. The spectroscopic constants of XOH are calculated in Supplementary Materials [30].

### 2.2. Laser Cooling Scheme

Laser direct cooling of molecules relies on momentum transfer from photons to molecules via repeated cycles of photon absorption and spontaneous emission. The process typically employs a closed-cycle electronic transition, where a molecule undergoes excitation from a low-energy rovibronic ground state to a higher-energy electronically excited state, followed by spontaneous emission back to the ground state.

For Doppler cooling, counter-propagating laser beams are frequency-tuned slightly below the transition resonance (red-detuned) to exploit the Doppler effect: molecules moving toward a laser beam experience an upshifted photon frequency, enhancing absorption and subsequent momentum kicks opposing their motion. This dissipates kinetic energy, leading to sub-Kelvin temperatures.

Similarly, laser cooling of polyatomic molecules requires the construction of near-closed photocycle transitions, which requires a high degree of overlap of the wavefunctions between the ground and excited states. The energy-level-transition strengths are quantified by FCFs. The molecular geometries, normal coordinates, and normal modes obtained by geometry optimization and frequency calculations are used to evaluate the FCFs. The results of the calculations using the 4-zeta basis set at the SA-CASSCF level are shown in Table 1 and Table 2. The calculated results are consistent with other theoretical calculations and experimental results (Δr_X-O_ ≤ 0.03; Δ_f_ ≤ 50 cm^−1^).

The results of FCF calculations from the ground state to the first excited state and the second excited state are shown in Table 3, where the FCFs are expressed as the square of the overlap integrals of the two vibrational wavefunctions since the ground- and excited-state vibrational modes are parallel and the Duschinsky rotations are considered. The FCFs for the transition listed in Table 3 are diagonalized. For the molecules CaOH and SrOH, the calculation results are in agreement with the experimental data, and the comparison results are presented in the Supplementary Materials. Geometry optimization results show that XOH is a linear molecule possessing four vibrational modes, including *v*_1_ (X-O stretching), *v*_2_ (X-O-H two degenerate bending modes), and *v*_3_ (H-O stretching), respectively. ${\tilde{X}}^{2}\Sigma_{1/2}^{+}(000)$ represents the ground vibrational state. The dominant FCFs for the XOH series (X = Ca, Sr, Ba) all surpass 0.9, where BaOH demonstrates exceptional vibronic coupling strength with its FCF reaching 0.988, significantly outperforming the CaOH and SrOH counterparts. The first three sum to greater than 0.99 for the ${X^{2}\Sigma }_{1/2}^{+}$ → $A^{2}\Pi_{1/2}$ transition. The ${X^{2}\Sigma }_{1/2}^{+}\to {B^{2}\Sigma }_{1/2}^{+}$ transition also possesses large FCFs. However, the sum of the FCFs is less sensitive to the small uncertainty of the bond-length difference [37]. Thus, some uncertainties of geometries are not expected to influence the efficiency of the proposed laser cooling schemes.

**Figure 4:**
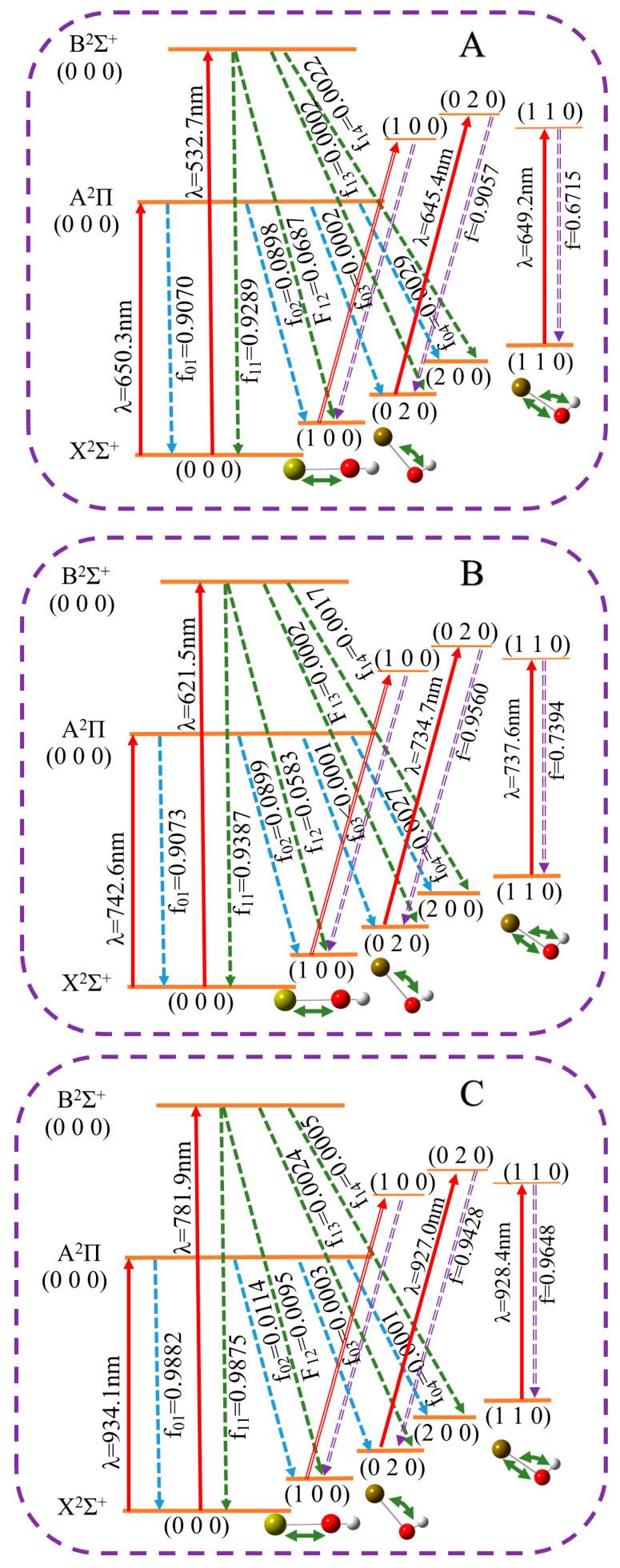
Proposed laser cooling and trapping scheme for XOH based on the SA-CASSCF calculation. (**A**–**C**) represent CaOH, SrOH, and BaOH molecular laser cooling processes, respectively, where electronic states with different vibrational modes are involved in laser repumping. Three different laser cooling schemes are provided for three molecules, along with corresponding data for experimentally limiting other vibrational dynamic branching ratios. The different vibrational energy levels are characterized by H = *v*_1_*v*_2_*v*_3_, where the three vibrational modes are *v*_1_ (X-O stretching), *v*_2_ (X-O-H bending), and *v*_3_ (H-O stretching). The dashed lines indicate the leap of FCFs from different ground-state energy levels to excited-state energy levels, and the red solid line represents the transition laser wavelength.

Theoretical analyses [38] and experimental studies [39] have consistently demonstrated that when multiple ground-state sublevels $n_{v_{g}}$ from different vibrational levels are coherently coupled to common excited-state sublevels $n_{0_{e}}$, the molecular scattering rate becomes suppressed by a factor of $2n_{0_{e}}/(n_{0_{e}}+\sum_{v}n_{v_{g}})$. This suppression mechanism consequently reduces the radiation pressure force. To maximize the scattering rate, the optimal configuration requires complete spectral decoupling of all repump lasers from the primary cycling transition. Based on this principle, we developed a laser cooling strategy featuring a primary cycling transition between the ${X^{2}\Sigma }_{1/2}^{+}$ and $A^{2}\Pi_{1/2}$ electronic states in Figure 4. To ensure comprehensive vibrational mode suppression, the system incorporates four auxiliary laser beams in addition to the primary cooling laser. These auxiliary beams are spectrally tailored to address specific vibrational excitations: one beam targeting the X–O stretching vibrational modes (*v*1 ≥ 0), one beam addressing the X–O–H bending vibrational modes (*v*2 > 0), and two hybrid beams simultaneously suppressing stretch–bend-coupled vibrational modes (*v*1, *v*2 > 0). This five-laser architecture (one primary and four auxiliary) achieves simultaneous electronic cycling and vibrational state control, significantly enhancing cooling efficiency across all degrees of freedom.

**Figure 5:**
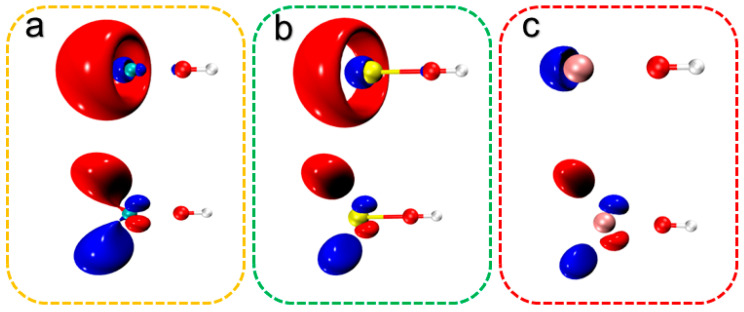
Frontier molecular orbitals of XOH, where (**a**–**c**) show HOMO and LUMO for CaOH, SrOH, and BaOH, respectively (isovalue: 0.05).

The XOH (X = Ca, Sr, Ba) molecules consist of an alkaline-earth metal with a hydroxyl group and an unpaired electron in the central atom. The highest occupied and lowest unoccupied orbitals of XOH (X = Ca, Sr, Ba) are shown in Figure 5, from which the electron transitions occur in the bonding region (near the optical cycling centers) during the laser cooling process, and the non-bonding electrons have almost no effect on the transitions. For XOH (X = Ca, Sr, Ba), the electronic structure of the first excited state is mainly generated by the *sσ*-orbital to *pπ*-orbital transitions of the valence electrons.

### 2.3. Laser Cooling Feasibility Assessment

To evaluate the feasibility of the laser cooling scheme, we implemented a Markov chain model following the methodology in Reference [18], where spontaneous emission probabilities were assigned based on the vibrational branching ratios (VBRs) between excited and ground electronic states. The optical excitation was restricted to the (0,0,0) vibrational states of both electronic manifolds, while adjacent vibrational modes (100), (020), and (200) remained spectroscopically dark under our pumping conditions. The VBRs were calculated through the Franck–Condon formulation:

$$b_{v^{\prime }v^{\prime \prime }}=\frac{f_{v^{\prime }v^{\prime \prime }}{\left(\Delta E_{v^{\prime }v^{\prime \prime }}\right)}^{3}}{\sum_{k}\;f_{v^{\prime }k}{\left(\Delta E_{vk^{\prime }}\right)}^{3}},$$

where $v^{\prime }$ and $v^{\prime \prime }$ denote vibrational quantum numbers in the ground (X) and excited (A) electronic states, respectively. $f_{v^{\prime }v^{\prime \prime }}$ represents the FCF, and $\Delta E_{v^{\prime }v^{\prime \prime }}$ corresponds to the vibronic energy gap.

The calculations predict remarkably high scattering counts: over 10,000 photons per molecular cycle for CaOH and SrOH, exceeding 15,000 for BaOH. These values indicate >99% closure fidelity for the optical cycling transitions, meeting the critical requirement for effective Doppler cooling. Notably, BaOH demonstrates enhanced technical feasibility through two synergistic effects: (1) reduced laser requirements for Stark deceleration due to its larger mass, and (2) simplified magneto-optical trapping configurations enabled by favorable branching ratios in the A-X band.

## 3. Methods

In this paper, we conduct a comprehensive study of XOH (X = Ca, Sr, Ba) molecules, utilizing accurate ab initio calculations. All single-point energy calculations are performed using the Complete Active Space Self-Consistent Field (CASSCF) [40] approach, which is subsequently followed by the Internally Contracted Multireference Configuration Interaction (icMRCI + Q) [41,42] method, augmented with the Davidson correction to refine the computational accuracy. Extensive testing was carried out. For oxygen and hydrogen, the aug-cc-pV5Z [43] basis set was employed to accurately describe electron correlation, polarization, and long-range interactions, with its 5-ζ quality ensuring the precise characterization of bond lengths. Scalar relativistic effects [44] in calcium were addressed using the Def2-QZVPPD [45] basis set, while spin–orbit relativistic effects in strontium and barium were incorporated via the dhf-QZVPP [46] basis set. These large basis sets maintain enhanced computational accuracy while accounting for relativistic contributions in heavy elements. The spin–orbit coupling (SOC) [47] is included in the MRCI calculation of electronic properties, producing a giant spin–orbit-induced splitting through the effective core potential (ECP) spin–orbit operator. The entire SO matrix elements are calculated and diagonalized by using the Breit–Pauli operator. All calculations of XOH are performed in the C_2v_ point group and carried out with the MOLPRO 2022.3 program [48].

Geometry optimization and normal mode analyses are performed by state-averaged CASSCF calculations without symmetry for XOH molecules. Calculation involving nine electrons distributed into an active orbital space of eight σ, six π, and one δ orbital, arising from the 5*d*, 6*s*, 6*p*, and 7*s* orbitals of barium, the 5*s*, 6*s*, 5*p*, and 4*d* orbitals of strontium, the 4*s*, 5*s*, 4*p*, and 3*d* orbitals of calcium, the 2*s* and 2*p* orbitals of oxygen, and the 1*s* orbital of hydrogen. The Franck–Condon factors were calculated using ezFCF v1.2 software [49], taking into account the Duschinsky rotation [50].

## 4. Conclusions

Employing high-level ab initio quantum chemical methods, we systematically calculated the PECs for both Λ-S states and spin–orbit-coupled Ω states in group II hydroxide radicals (XOH, X = Ca, Sr, Ba). Through a systematic investigation of electronic structure and transition dipole moments, near-closed optical cycling transitions were designed, and Markov chain simulations predicted scattering rates exceeding 10^4^ photons per cycle (15,000 for BaOH), satisfying Doppler cooling requirements. Our theoretical framework establishes BaOH as the most promising candidate owing to its exceptional spin–vibronic coupling characteristics and superior photon cycling efficiency. These findings provide crucial spectroscopic parameters and selection rule analyses for the experimental realization of ultracold polyatomic molecules.

## Figures and Tables

**Table 1:** Optimized geometry (in units of Å) and normal vibrational modes (in units of cm^−1^) for CaOH.

State	Coordinate	T1 [31]	TD [32]	Measured [33]	CASSCF (Def2-qzvppd)
$\tilde{X}$	Ca-O	1.9698	1.9656	1.9746	2.004
	O-H	0.9644	0.9646	0.9562	0.9517
	∠Ca-O-H	180	180	180	179.97
$\tilde{A}$	Ca-O	1.9698	1.9652	1.9532	1.9803
	O-H	0.9648	0.9646	0.9572	0.9514
	∠Ca-O-H	180	180		179.97
	Mode (symmetry)				
$\tilde{X}$	Bend (Π)	356.72	344.89/345.29	352.93	400.7
	Ca–O stretch (Σ)	625.22	603.84	609.02	625.59
	O–H stretch (Σ)	3816.28	3819.96	3778	3933.55
$\tilde{A}$	Bend (Π)	368.41	370.37/371.13	361.36	370.45
	Ca–O stretch (Σ)	625.18	603.84	630.68	642.44
	O–H stretch (Σ)	3816.06	3819.96		3960.06

**Table 2:** Optimized geometry (in units of Å) and normal vibrational modes (in units of cm^−1^) for BaOH and SrOH.

		Ba		Sr	
State	Coordinate	Measured [34]	CASSCF (Def2-qzvppd)	Measured [35,36]	CASSCF (Def2-qzvppd)
$\tilde{X}$	X-O	2.201	2.2539	2.111	2.17875
	O-H	0.927	0.9592	0.922	0.95134
	X-O-H		179.71		179.997
$\tilde{A}$	X-O	2.237	2.3171	2.091	2.12221
	O-H	0.758	0.9352	0.922	0.95047
	X-O-H		180		179.996
$\tilde{B}$	X-O	2.231	2.3171	2.098	2.12221
	O-H	0.909	0.9352	0.921	0.95047
	X-O-H		180		
	Mode (symmetry)				
$\tilde{X}$	Bend (Π)	341.6	355.44		387.6/387.6
	X–O stretch (Σ)	492.4	471.77		463.88
	O–H stretch (Σ)		3894.54		3935.41
$\tilde{A}$	Bend (Π)		381.69		399
	X–O stretch (Σ)		468.3		540.72
	O–H stretch (Σ)		3897.64		3960.14
$\tilde{B}$	Bend (Π)		399.63		421.17
	X–O stretch (Σ)		467.59		540.72
	O–H stretch (Σ)		3897.3		3960.14

**Table 3:** The calculated FCFs of ${X^{2}\Sigma }_{1/2}^{+}$ → $A^{2}\Pi_{1/2}$ and ${X^{2}\Sigma }_{1/2}^{+}\to {B^{2}\Sigma }_{1/2}^{+}$ transitions for XOH (X = Ca, Sr, Ba).

${{\tilde{X}}^{2}\Sigma }_{1/2}^{+}\left(\nu_{1}\nu_{2}\nu_{3}\right)\to {\tilde{A}}^{2}\Pi_{1/2}\left(\nu_{1}\nu_{2}\nu_{3}\right)$	FCF	Sum	${{\tilde{X}}^{2}\Sigma }_{1/2}^{+}\left(\nu_{1}\nu_{2}\nu_{3}\right)\to {{\tilde{B}}^{2}\Sigma }_{1/2}^{+}\left(000\right)$	FCF	Sum
CaOH					
${{\tilde{X}}^{2}\Sigma }_{1/2}^{+}\left(000\right)\to {\tilde{A}}^{2}\Pi_{1/2}\left(000\right)$	$9.07\times 10^{-1}$	0.9070	${{\tilde{X}}^{2}\Sigma }_{1/2}^{+}\left(000\right)\to {{\tilde{B}}^{2}\Sigma }_{1/2}^{+}\left(000\right)$	$9.299\times 10^{-1}$	0.9289
${{\tilde{X}}^{2}\Sigma }_{1/2}^{+}\left(100\right)\to {\tilde{A}}^{2}\Pi_{1/2}\left(000\right)$	$8.98\times 10^{-2}$	0.9968	${{\tilde{X}}^{2}\Sigma }_{1/2}^{+}\left(100\right)\to {{\tilde{B}}^{2}\Sigma }_{1/2}^{+}\left(000\right)$	$6.87\times 10^{-2}$	0.9976
${{\tilde{X}}^{2}\Sigma }_{1/2}^{+}\left(020\right)\to {\tilde{A}}^{2}\Pi_{1/2}\left(000\right)$	$2.00\times 10^{-4}$	0.9970	${{\tilde{X}}^{2}\Sigma }_{1/2}^{+}\left(020\right)\to {{\tilde{B}}^{2}\Sigma }_{1/2}^{+}\left(000\right)$	$2.00\times 10^{-4}$	0.9978
${{\tilde{X}}^{2}\Sigma }_{1/2}^{+}\left(200\right)\to {\tilde{A}}^{2}\Pi_{1/2}\left(000\right)$	$2.90\times 10^{-3}$	0.9999	${{\tilde{X}}^{2}\Sigma }_{1/2}^{+}\left(200\right)\to {{\tilde{B}}^{2}\Sigma }_{1/2}^{+}\left(000\right)$	$2.20\times 10^{-3}$	0.9998
${{\tilde{X}}^{2}\Sigma }_{1/2}^{+}\left(100\right)\to {\tilde{A}}^{2}\Pi_{1/2}\left(100\right)$	$7.39\times 10^{-1}$				
${{\tilde{X}}^{2}\Sigma }_{1/2}^{+}\left(020\right)\to {\tilde{A}}^{2}\Pi_{1/2}\left(020\right)$	$9.06\times 10^{-1}$				
SrOH					
${{\tilde{X}}^{2}\Sigma }_{1/2}^{+}\left(000\right)\to {\tilde{A}}^{2}\Pi_{1/2}\left(000\right)$	$9.07\times 10^{-1}$	0.9073	${{\tilde{X}}^{2}\Sigma }_{1/2}^{+}\left(000\right)\to {{\tilde{B}}^{2}\Sigma }_{1/2}^{+}\left(000\right)$	$9.39\times 10^{-1}$	0.9387
${{\tilde{X}}^{2}\Sigma }_{1/2}^{+}\left(100\right)\to {\tilde{A}}^{2}\Pi_{1/2}\left(000\right)$	$8.99\times 10^{-2}$	0.9972	${{\tilde{X}}^{2}\Sigma }_{1/2}^{+}\left(100\right)\to {{\tilde{B}}^{2}\Sigma }_{1/2}^{+}\left(000\right)$	$5.83\times 10^{-2}$	0.9970
${{\tilde{X}}^{2}\Sigma }_{1/2}^{+}\left(020\right)\to {\tilde{A}}^{2}\Pi_{1/2}\left(000\right)$	$1.00\times 10^{-4}$	0.9673	${{\tilde{X}}^{2}\Sigma }_{1/2}^{+}\left(010\right)\to {{\tilde{B}}^{2}\Sigma }_{1/2}^{+}\left(000\right)$	$2.0\times 10^{-4}$	0.9972
${{\tilde{X}}^{2}\Sigma }_{1/2}^{+}\left(200\right)\to {\tilde{A}}^{2}\Pi_{1/2}\left(000\right)$	$2.70\times 10^{-3}$	0.9999	${{\tilde{X}}^{2}\Sigma }_{1/2}^{+}\left(200\right)\to {{\tilde{B}}^{2}\Sigma }_{1/2}^{+}\left(000\right)$	$1.7\times 10^{-3}$	0.9999
${{\tilde{X}}^{2}\Sigma }_{1/2}^{+}\left(100\right)\to {\tilde{A}}^{2}\Pi_{1/2}\left(100\right)$	$8.73\times 10^{-1}$				
${{\tilde{X}}^{2}\Sigma }_{1/2}^{+}\left(020\right)\to {\tilde{A}}^{2}\Pi_{1/2}\left(020\right)$	$9.56\times 10^{-1}$				
BaOH					
${{\tilde{X}}^{2}\Sigma }_{1/2}^{+}\left(000\right)\to {\tilde{A}}^{2}\Pi_{1/2}\left(000\right)$	$9.88\times 10^{-1}$	0.9882	${{\tilde{X}}^{2}\Sigma }_{1/2}^{+}\left(000\right)\to {{\tilde{B}}^{2}\Sigma }_{1/2}^{+}\left(000\right)$	$9.88\times 10^{-1}$	0.9875
${{\tilde{X}}^{2}\Sigma }_{1/2}^{+}\left(100\right)\to {\tilde{A}}^{2}\Pi_{1/2}\left(000\right)$	$1.14\times 10^{-2}$	0.9996	${{\tilde{X}}^{2}\Sigma }_{1/2}^{+}\left(000\right)\to {{\tilde{B}}^{2}\Sigma }_{1/2}^{+}\left(000\right)$	$9.5\times 10^{-3}$	0.9970
${{\tilde{X}}^{2}\Sigma }_{1/2}^{+}\left(200\right)\to {\tilde{A}}^{2}\Pi_{1/2}\left(000\right)$	$3.0\times 10^{-4}$	0.9999	${{\tilde{X}}^{2}\Sigma }_{1/2}^{+}\left(200\right)\to {{\tilde{B}}^{2}\Sigma }_{1/2}^{+}\left(000\right)$	$2.4\times 10^{-3}$	0.9994
${{\tilde{X}}^{2}\Sigma }_{1/2}^{+}\left(020\right)\to {\tilde{A}}^{2}\Pi_{1/2}\left(000\right)$	$1.0\times 10^{-4}$	0.9999	${{\tilde{X}}^{2}\Sigma }_{1/2}^{+}\left(020\right)\to {{\tilde{B}}^{2}\Sigma }_{1/2}^{+}\left(000\right)$	$5.0\times 10^{-4}$	0.9999
${{\tilde{X}}^{2}\Sigma }_{1/2}^{+}\left(100\right)\to {\tilde{A}}^{2}\Pi_{1/2}\left(100\right)$	$9.65\times 10^{-1}$				
${{\tilde{X}}^{2}\Sigma }_{1/2}^{+}\left(020\right)\to {\tilde{A}}^{2}\Pi_{1/2}\left(020\right)$	$9.43\times 10^{-1}$				

## Data Availability

The data presented in this study are available upon request from the corresponding author.

## Supplementary Materials

The following supporting information can be downloaded at https://www.mdpi.com/article/10.3390/molecules30091950/s1: Figure S1: The transition dipole moment of the CaOH molecule; Figure S2: The transition dipole moment of the SrOH molecule; Figure S3: The transition dipole moment of the BaOH molecule; Table S1: Spectroscopic Constants of the CaOH Molecule; Table S2: Spectroscopic Constants of the SrOH Molecule; Table S3: Spectroscopic Constants of the BrOH Molecule; Table S4: CASSCF and MRCI Geometric Optimization and Frequency Calculations Compared with Experiments; Table S5: Transition information from the ground state to the excited state of CaOH; Table S6: Transition information from the ground state to the excited state of SrOH; Table S7: Transition information from the ground state to the excited state of BaOH; Table S8: Comparison results of transition information.
