# Supplementary material for: Production of Ultracold XOH (X = Ca, Sr, Ba) Molecules by Direct Laser Cooling: A Theoretical Study Based on Accurate Ab Initio Calculations
Source: Molecules. 2025 Apr 28;30(9):1950. doi: 10.3390/molecules30091950 (PMC12073337; doi:10.3390/molecules30091950)
Supplement: Supplementary file 1 [file molecules-30-01950-s001.zip › molecules-3552109-supplementary.pdf]

Production of ultracold XOH (X=Ca, Sr, Ba) molecules by direct laser cooling: A theoretical study based on accurate *ab initio* calculations

Jingbo Wei[1], Peng Li[1,2*], Jizhou Wu[1,2], Wenliang Liu[1,2], Yuqing Li[1,2], Yongming Fu[1,2], Jie Ma[1,2*]

[1] School of Physics and Electronic Engineering, State Key Laboratory of Quantum Optics and Quantum Optics Devices, Institute of Laser Spectroscopy, Shanxi University, Taiyuan 030006, China

[2] Collaborative Innovation Center of Extreme Optics, Shanxi University, Taiyuan 030006, China

*** Correspondence:**
Peng Li; Jie Ma
lip@sxu.edu.cn; mj@sxu.edu.cn

Table S1. Spectroscopic Constants of the CaOH Molecule

Table S2. Spectroscopic Constants of the SrOH Molecule

Table S3. Spectroscopic Constants of the BrOH Molecule

Fig S1 The transition dipole moment of the CaOH molecule.

Fig S2 The transition dipole moment of the SrOH molecule.

Fig S3 The transition dipole moment of the BaOH molecule.

Table S4. CASSCF and MRCI Geometric Optimization and Frequency Calculations Compared with Experiments

Table S5. Transition information from the ground state to the excited state of CaOH.

Table S6. Transition information from the ground state to the excited state of SrOH.

Table S7. Transition information from the ground state to the excited state of BaOH.

Table S8. Comparison results of transition information.

Table S1. Spectroscopic Constants of the CaOH Molecule

state	Re(Å)	Te(cm^{-1})	ωe(cm^{-1})	ωeχe(cm^{-1})	Be(cm^{-1})	ref
$\tilde{X}^2\Sigma^+$	2.0673	0	2984.975	60.4713	8.0674	this work
		0				Expt[1]
$\tilde{B}^2\Sigma^+$	2.044	18299.82	2994.763	63.6864	8.2541	this work
		18022.27				Expt
$\tilde{C}^2\Sigma^+$	2.3137	25992.85				this work
		25583.14				Expt
$\tilde{A}^2\Pi$	2.0441	15491.75	3079.544	62.1386	8.2524	this work
$\tilde{D}^2\Pi$	2.0602	32393.45	2528.739	51.7801	8.0959	this work

Table S2. Spectroscopic Constants of the SrOH Molecule

state	R_e(Å)	T_e(cm^{-1})	ω_e(cm^{-1})	$\omega_e\chi_e$(cm^{-1})	B_e(cm^{-1})	ref
$\tilde{X}^2\Sigma^+$	2.351	0	2222.569	31.383	6.2788	this work
	2.111	0				Expt[2]
$\tilde{B}^2\Sigma^+$	2.329	16129.91	2211.597	29.601	6.4086	this work
	2.098	16377.51				Expt[2]
$\tilde{C}^2\Sigma^+$	2.368	19854.81	2154.012	35.195	6.1903	this work
$\tilde{A}^2\Pi$	2.322	14211.98	2235.795	26.831	6.4487	this work
	2.091	14674.04				Expt[2]
$\tilde{D}^2\Pi$	2.367	27651.17	2060.553	31.998	6.2048	this work
	2.096	27307.25				Expt[2]

Table S3. Spectroscopic Constants of the BrOH Molecule

state	R_e(Å)	T_e(cm^{-1})	ω_e(cm^{-1})	$\omega_e\chi_e$(cm^{-1})	B_e(cm^{-1})	ref
$\tilde{X}^2\Sigma^+$	2.2128	0	2800.099	65.4416	7.0487	this work
	2.2	0				Expt[3]
$\tilde{B}^2\Sigma^+$	2.2357	12514.02	2147.356	80.6267	6.9119	this work
	2.231					Expt[3]
$\tilde{C}^2\Sigma^+$	2.2297	12548.35	2754.849	92.7601	6.9330	this work
$\tilde{A}^2\Pi$	2.2095	11037.71	2373.481	90.4627	7.0708	this work
	2.237	11483.44				Expt[3]
$\tilde{D}^2\Pi$	2.2282	20751.11	2357.338	86.4735	6.9491	this work

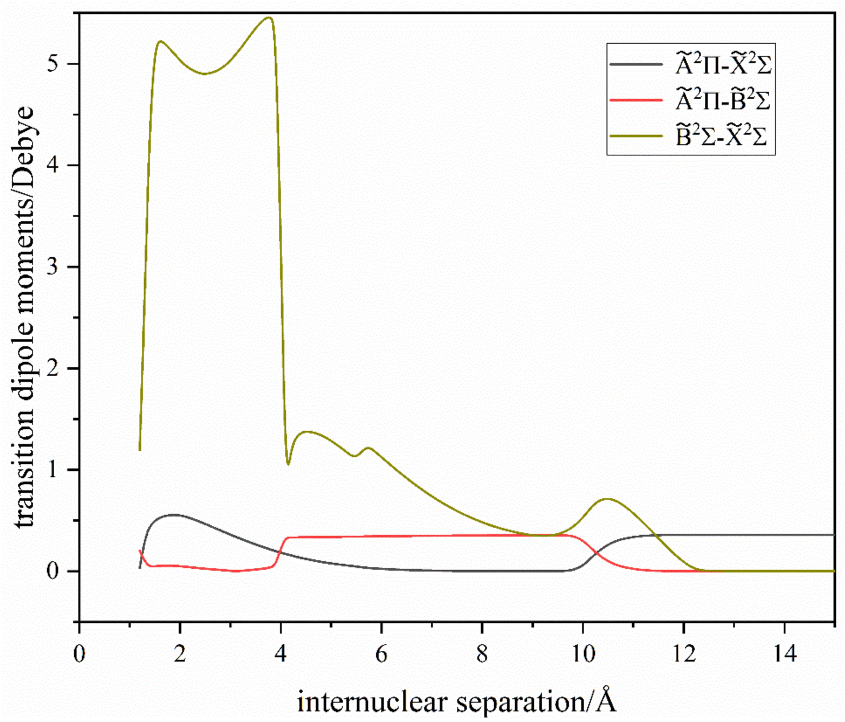

Fig S1 The transition dipole moment of the CaOH molecule.

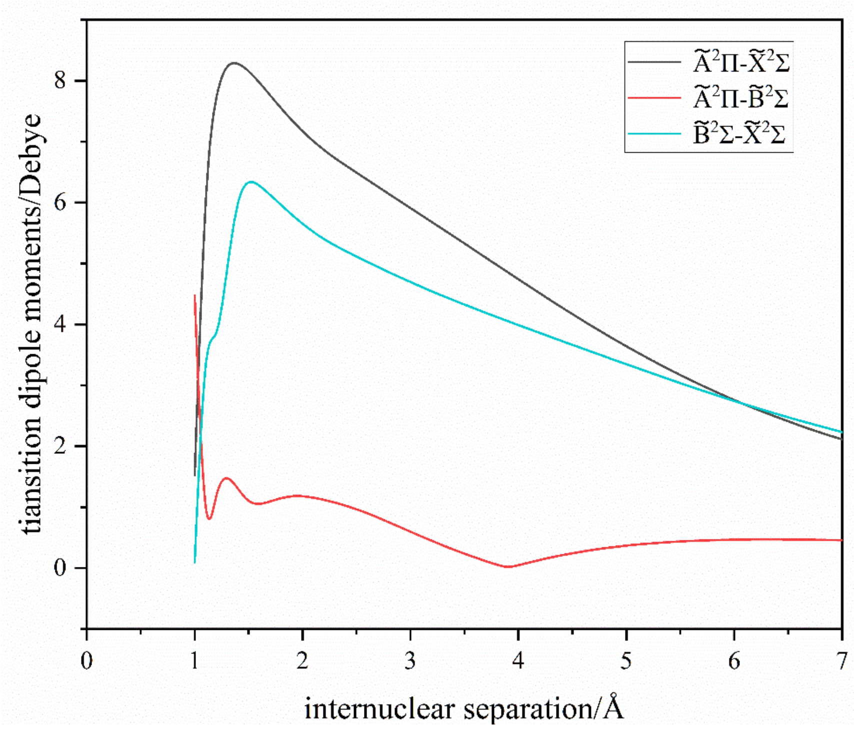

Fig S2 The transition dipole moment of the SrOH molecule.

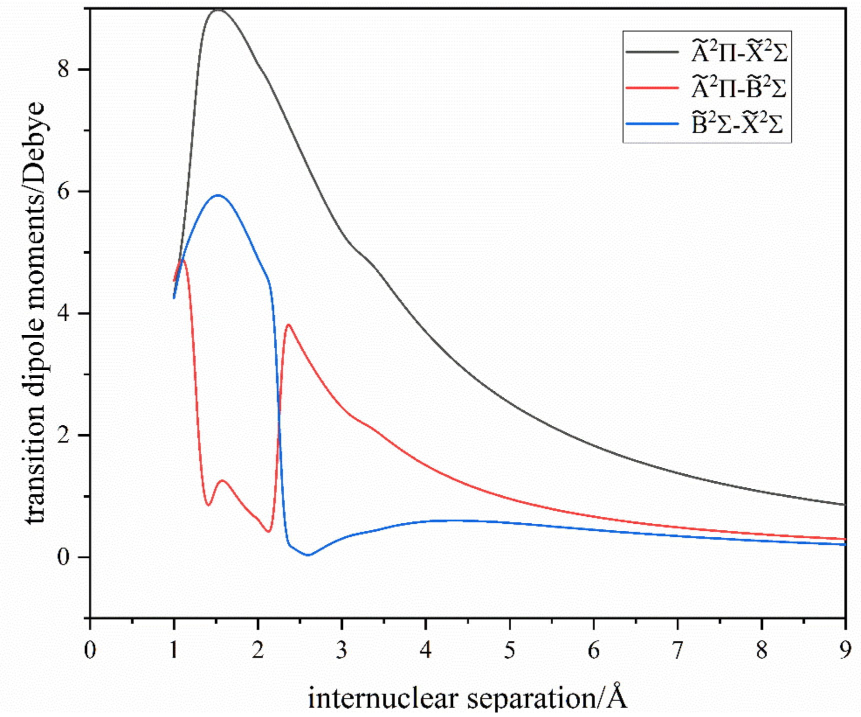

Fig S3 The transition dipole moment of the BaOH molecule.

Table S4. CASSCF and MRCI Geometric Optimization and Frequency Calculations Compared with Experiments

		Measured		CASSCF		MRCI	
	Coordinate	geometry	modes	geometry	modes	geometry	modes
Ca	X-O	1.9746	400.7	2.004	625.59	2.01988	620.59
	O-H	0.9562	3778	0.9517	3933.55	0.93221	4296.35
	X-O-H	180	352.93	179.97	400.7	179.98	413.08
Sr	X-O	2.111		2.17875	463.88	2.15002	512.97
	O-H	0.922		0.95134	3935.41	0.96207	4272.64
	X-O-H			179.997	387.6/387.6	180	448.31
Ba	X-O	2.201	492.4	2.2539	471.77	2.2446	473.11
	O-H	0.927		0.9592	3894.54	0.93497	4243.12
	X-O-H		341.6	179.71	355.44	179.734	415.87

Table S5. Transition information from the ground state to the excited state of CaOH.

CaOH		
X→A	FCF	Frequency
(020)→(000)	0.0004	597.1
(100)→(000)	0.072	606.68
(110)→(010)	0.0719	608.59
(000)→(000)	0.926	656.3
(010)→(010)	0.9256	658.2
(100)→(100)	0.7912	659.32
(020)→(020)	0.9238	660.04

(000)→(100)	0.0261	708.94
(000)→(020)	0.0004	719.24
X→B	FCF	Frequency
(010)→(000)	0.0737	514.31
(000)→(000)	0.9249	556.37
(100)→(100)	0.9243	557.65
(010)→(010)	0.7866	558.93
(200)→(200)	0.9231	558.98
(000)→(010)	0.0694	600.99

Table S6. Transition information from the ground state to the excited state of SrOH.

SrOH		
X→A	FCF	Frequency
(020)→(000)	0.0019	649.85
(200)→(000)	0.0004	677.03
(010)→(000)	0.0881	696.26
(110)→(100)	0.0878	698.19
(020)→(010)	0.1651	699.38
(100)→(000)	0.0004	709.85
(200)→(100)	0.0008	711.78
(010)→(100)	0.0002	730.94
(000)→(000)	0.9091	742.60
(100)→(100)	0.9073	744.53
(010)→(010)	0.7456	745.72
(200)→(200)	0.9048	746.46
(110)→(110)	0.7440	747.65
(020)→(020)	0.6044	748.84
(000)→(100)	0.0003	777.35
(100)→(200)	0.0006	779.28
(010)→(110)	0.0002	780.40
(200)→(300)	0.0008	781.22
(000)→(010)	0.0826	792.06
(100)→(110)	0.0825	793.99
(010)→(020)	0.1447	795.18
(200)→(210)	0.0823	795.99
(110)→(120)	0.1445	797.11
(010)→(030)	0.1891	798.30
(000)→(200)	0.0004	812.03
(100)→(300)	0.0011	813.96
(010)→(210)	0.0003	815.15
(000)→(110)	0.0000	826.81
(100)→(210)	0.0001	828.74
(000)→(020)	0.0069	841.51
(100)→(120)	0.0069	843.52

(010)→(030)	0.0188	844.63
(000)→(210)	0.0000	861.49
(000)→(030)	0.0005	891.05
(100)→(001)	0.0002	1066.30
(200)→(101)	0.0003	1068.30
(000)→(101)	0.0001	1133.80
(100)→(201)	0.0002	1135.73
(000)→(111)	0.0000	1183.26
X→B	FCF	Frequency
(010)→(000)	0.0571	582.7184
(110)→(100)	0.0570	583.3399
(020)→(010)	0.1103	584.7694
(100)→(000)	0.0004	594.0919
(200)→(100)	0.0008	594.7134
(010)→(100)	0.0002	610.8102
(000)→(000)	0.9417	621.5
(100)→(100)	0.9404	622.1837
(200)→(200)	0.9390	622.8052
(010)→(010)	0.8329	623.551
(110)→(110)	0.8317	624.2346
(020)→(020)	0.7334	625.6019
(000)→(100)	0.0003	649.5918
(100)→(200)	0.0006	650.2755
(200)→(300)	0.0009	650.9591
(010)→(110)	0.0002	651.6428
(000)→(010)	0.0543	662.3947
(100)→(110)	0.0543	663.0162
(200)→(210)	0.0542	663.6999
(010)→(020)	0.0994	664.4457
(110)→(120)	0.0993	665.1293
(020)→(030)	0.1362	666.4966
(000)→(200)	0.0001	677.6836
(100)→(300)	0.0002	678.3673
(000)→(110)	0.0000	690.4865
(100)→(210)	0.0001	691.108
(000)→(020)	0.0033	703.2273
(100)→(120)	0.0033	703.9109
(010)→(030)	0.0092	705.2782
(000)→(030)	0.0002	744.122
(100)→(001)	0.0002	892.7848
(200)→(101)	0.0004	893.4684
(000)→(101)	0.0001	948.2847
(100)→(201)	0.0003	948.9684
(010)→(111)	0.0001	950.3978

Table S7. Transition information from the ground state to the excited state of BaOH.

BaOH		
X→A	FCF	Frequency
(010)→(000)	0.0152	879.8759
(110)→(010)	0.0152	880.1561
(020)→(100)	0.0304	881.1837
(000)→(000)	0.9847	934.15
(100)→(010)	0.9847	934.3368
(200)→(020)	0.9847	934.6171
(010)→(100)	0.9547	935.4578
(110)→(110)	0.9547	935.6446
(020)→(200)	0.9253	936.7656
(000)→(100)	0.0149	989.7319
(100)→(110)	0.0149	989.9188
(200)→(121)	0.0149	990.1056
(010)→(200)	0.0289	991.0397
(110)→(210)	0.0289	991.2266
(020)→(300)	0.0422	992.3475
(000)→(200)	0.0004	1045.22
(100)→(210)	0.0004	1045.501
(010)→(300)	0.0011	1046.528
X→B	FCF	Frequency
(200)→(000)	0.0009	709.138
(110)→(010)	0.0081	733.4535
(020)→(100)	0.0160	735.6427
(010)→(000)	0.0082	736.4245
(200)→(020)	0.9849	775.7516
(110)→(110)	0.9723	778.0189
(100)→(010)	0.9887	778.8008
(020)→(200)	0.9578	780.2081
(010)→(100)	0.9742	781.0682
(000)→(000)	0.9906	781.85
(200)→(120)	0.0083	820.317
(110)→(210)	0.0166	822.5844
(100)→(110)	0.0083	823.3662
(020)→(300)	0.0249	824.8518
(010)→(200)	0.0166	825.6336
(000)→(100)	0.0083	826.4155
(100)→(030)	0.0028	845.4144
(010)→(120)	0.0009	847.6818
(000)→(020)	0.0009	848.4636
(100)→(011)	0.0001	1158.233
(000)→(001)	0.0001	1161.282

Table S8. Comparison results of transition information.

SrOH				
	FCF		Frequency(cm^{-1})	
X→A	this work	ref	this work	Expt[4]
(000)→(000)	0.9091	0.9451	742.60	688
(020)→(010)	0.1651		699.38	697
X→B				
(010)→(000)	0.0082		736.42	631
(020)→(100)	0.0160		735.64	631
(110)→(010)	0.0081		733.4535	624
(200)→(000)	0.0009		709.138	638
**CaOH**				
	FCF		Frequency(cm^{-1})	
X→A	this work	ref	this work	Expt[5]
(000)→(000)	0.926	0.954	656.3	626
(020)→(000)	0.0004	0.0003	597.1	555

1 Bernath, P. F., and C. R. Brazier. "Spectroscopy of CaOH." Astrophysical Journal, Part 1 (ISSN 0004-637X), vol. 288, Jan. 1, 1985, p. 373-376. Research supported by the Petroleum Research Fund. 288 (1985): 373-376.

2 Wang, J. G., Dick, M. J., Sheridan, P. M., Yu, S., & Bernath, P. F. (2007). Further spectroscopic investigations of the high energy electronic states of SrOH: The $\tilde{B}^2\Sigma^+$ (000)–$\tilde{A}^2\Pi$ (000) and the $\tilde{D}^2\Sigma^+$ (000)–$\tilde{A}^2\Pi$ (000) transitions. Journal of Molecular Spectroscopy, 245(1): 26-33.

3 Tandy, J. D., Wang, J. G., & Bernath, P. F. (2009). High-resolution laser spectroscopy of BaOH and BaOD: Anomalous spin-orbit coupling in the $\tilde{A}^2\Pi$ state. *Journal of Molecular Spectroscopy*, *255*(1), 63-67.

4 Lasner, Z., Lunstad, A., Zhang, C., Cheng, L., & Doyle, J. M. (2022). Vibronic branching ratios for nearly closed rapid photon cycling of SrOH. Physical Review A, 106(2), L020801.

5 Kozyryev, I., Steimle, T. C., Yu, P., Nguyen, D. T., & Doyle, J. M. (2019). Determination of CaOH and CaOCH3 vibrational branching ratios for direct laser cooling and trapping. New Journal of Physics, 21(5), 052002.
